# Estrous Behaviour and Vulva Score in Captive Female Malayan Tapir (*Tapirus indicus*)

**DOI:** 10.21315/tlsr2025.36.1.10

**Published:** 2025-03-30

**Authors:** Donny Yawah, Jeffrine Rovie-Ryan, Azlan Che-Amat, Michelle Fong Wai Cheng, Hartini Ithnin, Mohd Adli Ahmad, Enos Jeoffry, Madzlan Misnan, Zaihamrezal Abdul Hamid, Mark WH Hiew

**Affiliations:** 1 Department of Veterinary Clinical Studies, Faculty of Veterinary Medicine, Universiti Putra Malaysia, 43400 Serdang, Selangor, Malaysia; 2 Department of Wildlife and National Parks (PERHILITAN), Peninsular Malaysia KM10, Jalan Cheras, 56100 Kuala Lumpur, Malaysia; 3 Faculty of Resource Science and Technology, Universiti Malaysia Sarawak, 94300 Kota Samarahan, Sarawak, Malaysia; 4 Sungai Dusun Wildlife Conservation Centre, Sungai Dusun, 44010 Kuala Kubu Bahru, Selangor, Malaysia

**Keywords:** Malayan Tapir, Estrus Behaviour, Vulva Score, Tapir Reproduction, Estrous Cycle, Tapir Malaya, Tingkah Laku Pembiakan, Skor Vulva, Pembiakan Tapir, Kitaran Estrus

## Abstract

The ex-situ propagation of the Malayan tapir in Malaysia is an important conservation strategy to increase the population of this endangered species. However, the scarcity of reproductive physiology studies impedes potential conservation efforts. This study aimed to investigate the expression of estrus signs through observation of estrus behaviour and vulva score in captive Malayan tapirs. This study was conducted from January to April at the Sungai Dusun Wildlife Conservation Centre, Selangor, Malaysia. A total of five female Malayan tapirs aged between 3 to 18 years old were involved in this study. Two of the tapirs were kept with a male partner, while three others were kept individually. Closed-circuit television (CCTV) cameras with night vision and audio recording function connected to a digital video recorder were used to record the female activity from 1930 to 0730 hours. The recorded videos were evaluated for estrus behaviour such as vocalisation, flehmen response, urine spraying and excretion smelling. The vulva score (scale of 0 to 4) was determined based on the vulva size and the presence of discharge. The most common estrus behaviour observed in the female Malayan tapirs was vocalisation (81%), followed by flehmen response (10%), urine spraying (5%) and excretion smelling (4%). The paired females were observed to exhibit more estrus behaviours than the non-paired females. Vulva changes were observed in both groups with evidence of cyclic pattern with an average median length of 31.0 days (range of 28 days to 33 days). Mann-Whitney U test revealed significant difference between paired group and non-paired group for vocalisation and urine spraying. Meanwhile, Spearman’s rho test showed a strong positive correlation between vulva score and flehmen response and very strong positive correlation between vulva score and excretion smelling. This study provides important information for the establishment of future captive propagation programmes.

## Abstract (fr)

**Abstrak:**

Pembiakan Tapir Malaya (*Tapirus indicus*) dalam kurungan di Malaysia merupakan strategi pemuliharaan penting untuk meningkatkan populasi spesies yang terancam ini. Namun, kekurangan kajian fisiologi pembiakan menghalang potensi usaha pemuliharaan. Kajian ini bertujuan menyiasat ekspresi tanda estrus melalui pemerhatian tingkah laku pembiakan dan skor vulva pada Tapir Malaya dalam kurungan. Kajian ini dilakukan dari Januari hingga April di Pusat Konservasi Hidupan Liar Sungai Dusun, Selangor, Malaysia. Sejumlah lima ekor Tapir Malaya betina berumur antara 3 hingga 18 tahun terlibat dalam kajian ini. Dua daripada tapir ditempatkan dalam kurungan berasingan bersama seekor jantan, manakala tiga lainnya disimpan secara individu. Kamera CCTV dilengkapi dengan penglihatan malam dan rakaman audio yang disambungkan ke perakam video digital digunakan untuk merakam aktiviti betina dari jam 1930 hingga 0730. Video yang direkodkan dinilai untuk tingkah laku pembiakan seperti vokalisasi, tindak balas flehmen, semburan air kencing, dan menghidu najis. Skor vulva (skala 0 hingga 4) ditentukan berdasarkan pembengkakan vulva dan kehadiran discaj. Kelakuan pembiakan paling banyak ditunjukkan adalah vokalisasi (81%), diikuti oleh tindak balas flehmen (10%), semburan air kencing (5%), dan menghidu najis (4%). Tapir betina yang mempunyai pasangan menunjukkan lebih banyak tingkah laku pembiakan berbanding tapir betina tanpa pasangan. Perubahan skor vulva didapati berlaku pada kedua-dua kumpulan tapir. Perubahan pada vulva diperhatikan berlaku pada kedua-dua kumpulan dengan bukti corak kitaran dengan purata panjang median 31.0 hari (julat antara 28 hari hingga 33 hari). Ujian Mann-Whitney U menunjukkan perbezaan yang signifikan antara kumpulan berpasangan dan kumpulan tidak berpasangan bagi vokalisasi dan penyemburan air kencing. Manakala, ujian Spearman’s rho menunjukkan korelasi positif yang kuat antara skor vulva dan tindak balas flehmen serta korelasi positif yang sangat kuat antara skor vulva dan menghidu najis. Kajian ini memberikan maklumat penting untuk penambahbaikan program pembiakan dalam kurungan pada masa hadapan.

**Highlights**

- Vulva changes were observed in both paired and non-paired females with evidence of cyclic pattern with an average median length of 31 days (range of 28 days to 33 days).
- Relying solely on the changes in estrous behaviour, especially within the non-paired females, does not provide reliable indication for the prediction of estrus in Malayan tapir.
- However, the combination of both estrous behaviour and vulva score allows for early prediction of the reproductive status in Malayan tapirs.

## INTRODUCTION

Malayan tapir (*Tapirus indicus*), also known as Asian tapir, is the largest of the four world tapir species. It is exclusively distributed in the Southeast Asian region which includes Peninsular Malaysia, the southern part of Myanmar and Thailand, and Sumatera, Indonesia. The Malayan tapir is endangered because of habitat destruction and fragmentation, being accidentally caught in snare wires and being victim of road kills (Holden *et al*. 2003; Corlett 2007). According to the International Union for Conservation of Nature (IUCN) Red List of Threatened Species, the estimate of the world tapir population is approximately 2,500 mature individuals (Traeholt *et al*. 2016). Malaysia has a population of between 1,100 and 1,500 individuals (PERHILITAN 2009). In Indonesia and Thailand, the tapir population is estimated to reach 400 to 500 (Traeholt *et al*. 2016) and 532 to 720 individuals, respectively (Kanchanasaka 2015). No population estimate exists for Myanmar.

The Malayan tapir is a solitary animal (Williams & Petrides 1980) but has been seen in pairs or groups around salt licks (Williams & Petrides 1980; Tawa *et al*. 2021), especially during the breeding season (William & Petrides 1980; Kusuda *et al*. 2007) or when a female is together with its offspring (Donny *et al*. 2022). In the wild, Malayan tapirs are nocturnal and are mostly active during the night (1900–0600 h) as well as occasionally during the daytime (0600–0800, 1000–1100 and 1600–1700 h) (Holden *et al*. 2003; Novarino & Grant 2005; Magintan *et al*. 2009; Khadijah-Ghani 2010; Marlius *et al*. 2018). Captive Malayan tapirs exhibit both nocturnal (Lynam *et al*. 2012; Suwannaphong *et al*. 2018) and diurnal (Gillmore 2007; Kusuda *et al*. 2008; Kamisan 2017; Suwannaphong *et al*. 2018; Arumugam *et al*. 2020) activity. In captivity, the Malayan tapir spends most of the day time resting (sleeping and lying), followed by eating, drinking, exploring and mating (Wazirotul *et al*. 2014; Suwannaphong *et al*. 2018). However, there are no reports on the night time activity patterns of captive Malayan tapirs. Sexual behaviours are more likely to be expressed at night or twilight rather than daytime (Novarino & Grant 2005; Lynam *et al*. 2012).

In a recent review of the technique for monitoring the estrous cycle in Malayan tapirs, Donny and Hiew (2024) highlighted significant gaps in our understanding of Malayan tapir reproductive physiology. The estrus behaviour patterns vary according to the different phases of the estrous cycle. According to Sankar Ganesh *et al*. (2014), females in estrus display distinct behaviours including increased restlessness, vocalisation, standing heat, vaginal discharge and decreased food intake. Schaftenaar *et al*. (2006) did not observe any typical estrus behaviours in their study of a captive Malayan tapir. In contrast, Kusuda *et al*. (2008) found that female Malayan tapirs exhibited increased vocalisation and urine squirting during the copulatory period compared to the non-copulatory period in their study of two different pairs. The smelling of excretion or external genitalia of a female which was often followed by flehmen response is a common way for a male mammal to determine a female in estrus (Campbell 2004) and this has been seen in tapirs (Kusuda *et al*. 2008). The flehmen response is manifested by dorso-flexion of the neck with eyes rolled back and the upper lip curled exposing the upper incisors and adjacent gums (Gillmore 2007). However, it is unknown whether these behaviours could indicate estrus although they have been occasionally observed by keepers at Sungai Dusun Wildlife Conservation Centre during mating season.

The swelling of the vulva accompanied by clear mucus discharge has been documented (Schaftenaar *et al*. 2006) alongside low progesterone (P4) concentrations (Kusuda *et al*. 2007). Hence, visual changes in the vulva act as real-time indicators of a female’s estrus (Kusuda *et al*. 2007). However, no research has been conducted on vulva appearance in relation to behaviour changes during estrus in Malayan tapirs. Both of these techniques could benefit the captive breeding management of Malayan tapirs. This study aimed to investigate the estrus behaviour pattern and vulva changes of captive female Malayan tapirs.

## MATERIALS AND METHODS

### Ethical Statement

This study was approved by the Institutional Animal Care and Use Committee of Universiti Putra Malaysia (Reference number: UPM/IACUC/AUP-R068/2020). It was completed in strict accordance with the relevant guidelines and regulations from the Department of Wildlife and National Parks Peninsular Malaysia (Permit number: B-00369-16-2).

### Study Site

**Figure 1:**
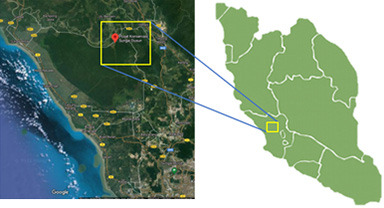
Location of Sungai Dusun Wildlife Conservation Centre. (*Source*: Google Earth Image)

**Figure 2:**
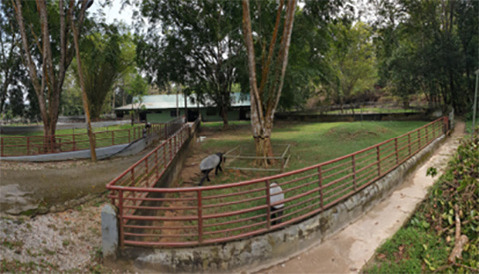
The central stable and outdoor enclosure at the Sungai Dusun Wildlife Conservation Centre.

This study was conducted at Sungai Dusun Wildlife Conservation Centre, in the state of Selangor, Malaysia (Fig. 1). The centre has 10 central nightstalls surrounded by seven outdoor enclosures which the animals have access to at all times of the day. Each nightstall has an area of approximately 20 m^2^ with feeding and drinking trays provided. The area of each outdoor enclosure is approximately 250 m^2^ and is composed of grass and several trees (Fig. 2). The outdoor enclosures are bordered by concrete walls and metal bars. The centre is used to shelter and provide veterinary care and treatment for rescued Malayan tapirs as well as for breeding in captivity.

### Animals

Five adult female Malayan tapirs aged between 3 and 18 years old were selected for this study (Table 1). Two of the tapirs (MT1, MT2) were kept with a male, while the other three (MT3, MT4 and MT5) were kept individually. Both males were proven bulls with a track record of producing offspring. All tapirs were kept in their own enclosures. The tapirs were fed similar diets which included leaves, fruits and commercial horse pellets. Clean and fresh water was provided *ad libitum*.

### Observation and Documentation of Estrus Behaviour

Ten closed-circuit television (CCTV) cameras (HDCVI Bullet Camera, Dahua Technology, Hangzhou) with night vision and audio recording functions were used to record each subject’s activity (two for each enclosure, one for nightstall and the other for the outdoor enclosure). The CCTV cameras were connected to a digital video recorder and set for continuous recording between 1930 h and 0730 h from January to April. The recorded video footage was retrieved weekly and evaluated for estrus behaviour according to an ethogram which included vocalisation, smelling of excretion, flehmen response and spraying urine (Table 2). Behaviour expressed by each subject was observed at every minute and the total was recorded as the daily frequency. Mounting behaviour of the paired group was also documented.

### Observation and Evaluation of Vulvar Changes

Evaluation of the vulva was performed with the tapir in a standing position, from approximately one meter posterior to the animal. Before the vulva was scored, the hindlimbs were made sure to be parallel to each other. The vulva was evaluated from the left, right and centre for any swelling and/or discharge. The final vulva score was recorded as the sum of the vulva swelling score and the vaginal discharge score (Table 3).

### Data Analysis

The daily estrus behaviour frequency and vulva score for each tapir were recorded and analysed for patterns and relationships. Mann-Whitney U test was used to compare the estrus behaviour of paired and non-paired groups. Meanwhile, Spearman’s correlation was used to determine the correlation between vulva score (when vulva score > 0) and each estrus behaviour. Both tests were run in IBM SPSS version 26 (IBM Corps, USA). *p <* 0.05 was considered statistically significant.

## RESULTS

### Estrus Behaviour

**Figure 3:**
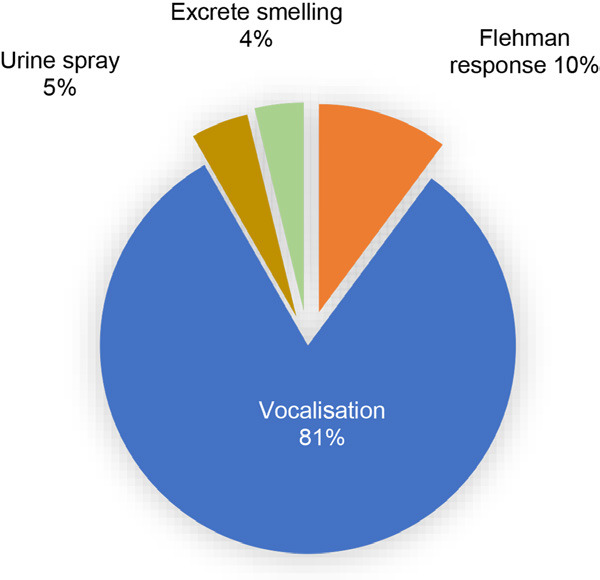
The breeding behaviours of five captive female Malayan tapirs at Sungai Dusun Wildlife Conservation Centre from January to April.

**Figure 4:**
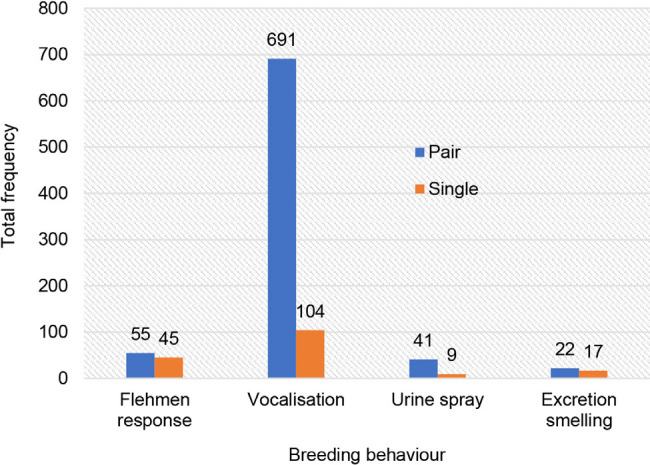
Frequency of breeding behaviours in captive Malayan tapirs housed in pairs or as single animals at Sungai Dusun Wildlife Conservation Centre from January to April.

The estrus behaviour of captive female Malayan tapir was dominated by vocalisation (81%), followed by flehmen response (10%), urine spraying (5%) and excretion smelling (4%) (Fig. 3). Tapirs housed in pairs (MT1 and MT2) exhibited a greater frequency of estrus behaviours than those housed individually (MT3, MT4 and MT5) (Fig. 4). The estrus behaviour patterns of each individual are shown in Fig. 6. Meanwhile, the mounting behaviour of the paired group (MT1 and MT2) was noted in the graphs in Figs. 6a and 6b.

### Vulva Score

**Figure 5:**
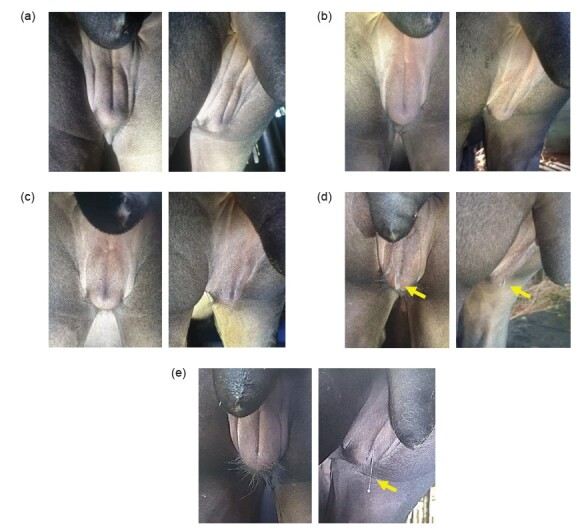
Comparison of vulva score of a Malayan tapir in this study (MT3). (a) Score 0; no enlargement and no discharge, (b) Score 1; slight enlargement of the vulva but no discharge, (c) Score 2; obvious enlargement of vulva without discharge, (d) Score 3: obvious enlargement of the vulva with small discharge from the vulva (arrow) and (e) Score 4: obvious enlargement of the vulva and copious clear and thick discharge (arrow).

**Figure 6:**
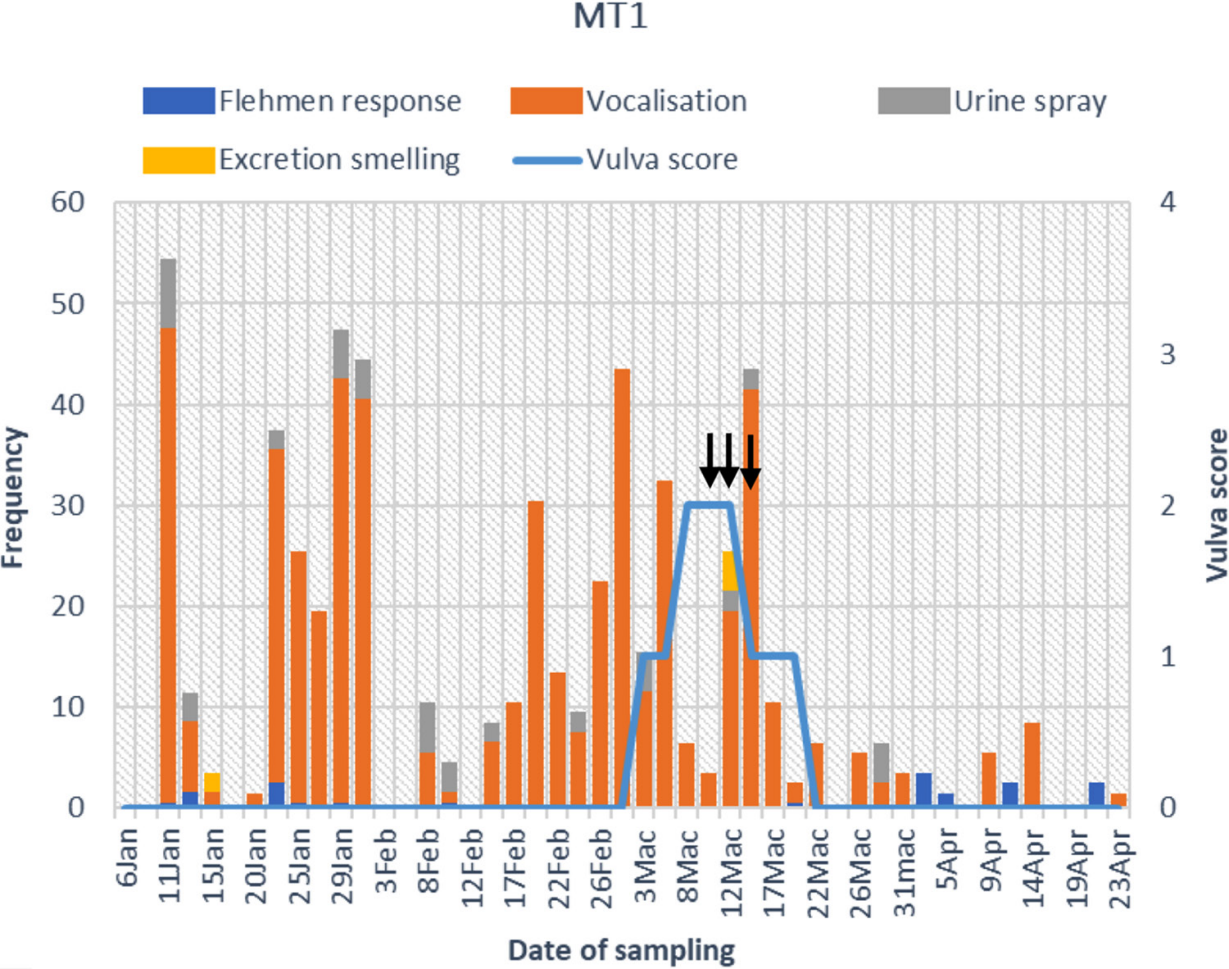

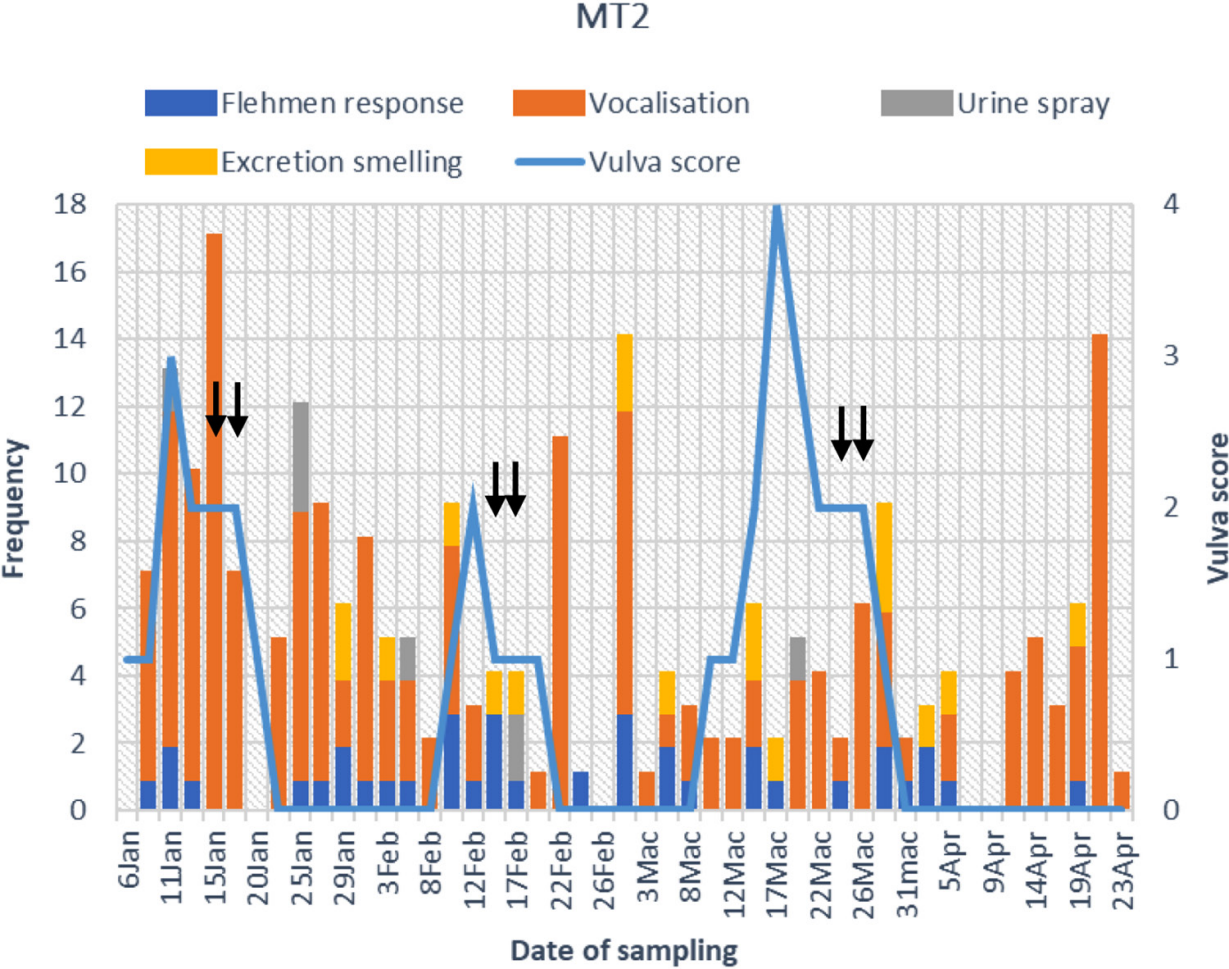

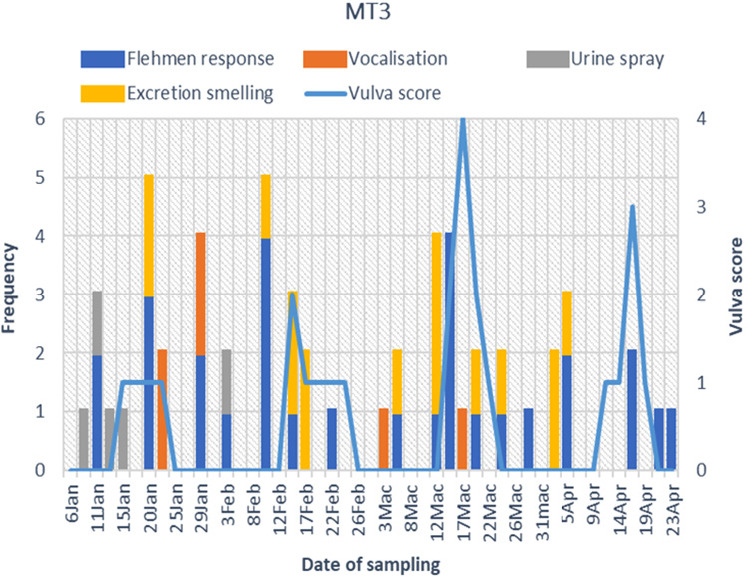

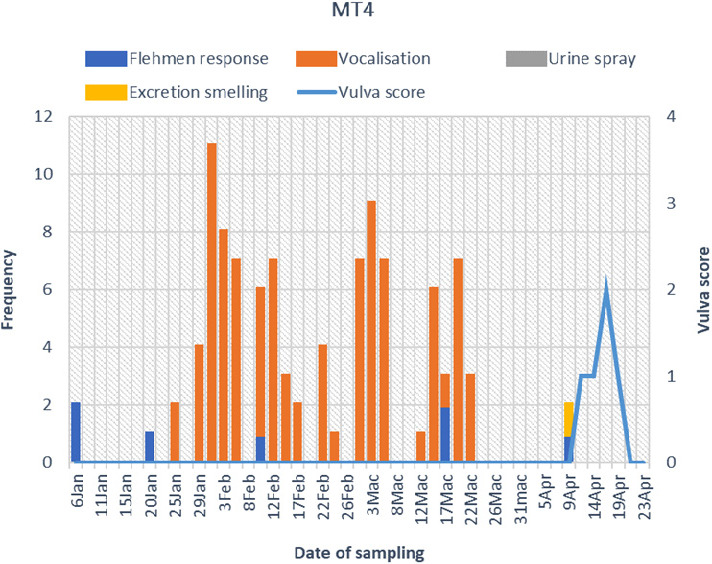

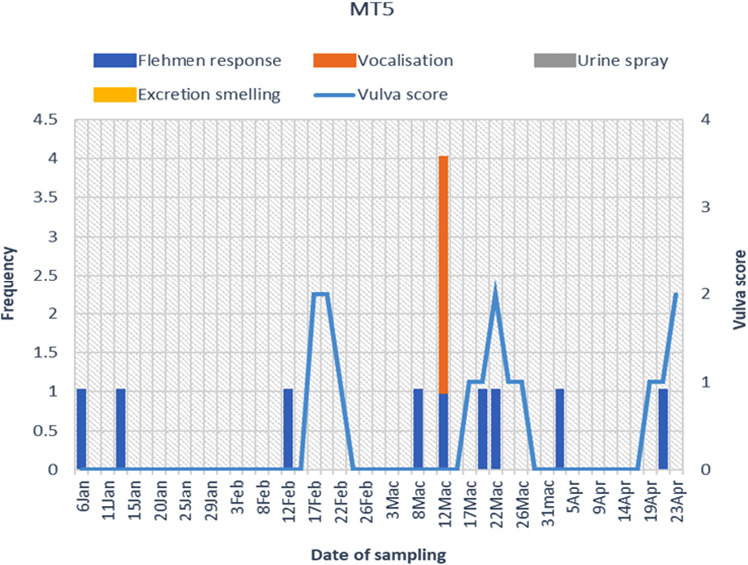
The frequency of breeding behaviour; flehmen response, vocalisation, urine spray and excretion smelling (bar graph), vulva score (line graph) and mounting behaviour (black arrows) of five captive female Malayan tapirs in Sungai Dusun Wildlife Conservation Centre from January to April for (a) MT1, (b) MT2, (c) MT3, (d) MT4 and (e) MT5.

Vulva scores of 0 to 4 were observed in this study (Fig. 5). A total of 12 events and 7 cycles associated with increased vulva scores were observed among the five Malayan tapirs. Based on the vulva scores, a cyclical pattern was observed in all tapirs (Fig. 6). The median and range of the duration and length of the increased vulva score were as follows: the duration had a median of 9 days and range of 4 to 16 days. Meanwhile the length had a median of 31 days and range of 28 to 33 days (Table 4).

### Mann-Whitney U Test for Significant Different Between Paired Group and Non-paired Group

Mann-Whitney U test was run to analyse the values of flehmen response, vocalisation, urine spraying and excretion smelling between the paired group and non-paired group (when vulva score > 0). The test revealed a significant difference for vocalisation (*U* = 0.00, *p =* 0.010, *Z =* −2.566) and urine spraying (*U* = 0.00, *p =* 0.008, *Z =* −2.640). However, there were no significant differences for flehmen response and excretion smelling (Table 5).

### Spearman Correlation Test for Vulva Score and Estrus Behaviour Camparison

A Spearman correlation test was conducted to compare vulva scores and estrus behaviour. The result indicates a strong positive correlation between vulva score and flehmen response (*r_s_* = 0.900, *n* = 5, *p* = 0.037) and very strong positive correlation between vulva score and excretion smelling (*r_s_* = 0.975, *n* = 5, *p =* 0.005). However, there were no significant correlations for vulva score with vocalisation and urine spraying (Table 6).

## DISCUSSION

Mammals express substantial variations in the display of behaviours during different reproductive stages. For many species in captivity, estrus signs are not easily observed. This is particularly due to changes in social, natural habits or enclosure conditions, stress caused by handling, and management (Santiago *et al*. 2007). In this study, the non-paired group expressed significantly less estrus behaviour compared to the paired group and this is possibly due to the lack of stimulation from a male. In terms of vocalisation, the females in the non-paired group showed fewer vocalisations than those in the paired group. This provides evidence that the presence of a male has a significant influence on the female. Females were seen to vocalise more when the males actively trailed and chased them during estrus.

A total of 100 flehmen responses were recorded from the five females over the course of the study with the paired group at 55 and the non-paired group at 45. The females in the paired group showed flehmen response after smelling the male’s excretion or external genitalia. Meanwhile, MT3 and MT5 showed flehmen response after smelling either their own excretion or excretion from the neighbouring tapir. Although flehmen response is a common way for a male mammal to determine a female in estrus (Campbell 2004), it is unknown whether flehmen response in the female tapir could indicate estrus. The occurrence of flehmen response in this study is infrequent and few (1–4 times within 12 h), making it an impractical method to determine estrus in Malayan tapirs.

Urine spraying is a natural marking behaviour for many mammals. The female tapirs in this study showed a total of 50 urine spraying events. The paired group displayed significantly more spraying (*n* = 41) compared to the non-paired group (*n* = 9) (Fig. 4). The female tapir’s urine spraying was significantly associated with the presence of the male in the same enclosure. They regularly sprayed urine at the male during mating attempts. This behaviour is likely essential for the female to signal her current physiological reproductive condition to the male either as an indication of estrus or as a deterrent if she is not receptive to mounting. Meanwhile, the non-paired tapirs sprayed urine into the air or to the wall, probably for similar reasons observed in the paired group. Smelling female excretion is a general behaviour for a male mammal to determine female estrus (Campbell 2004). However, it is unknown whether the smelling of excretion by the female tapir is associated with their physiological reproductive stages. Tapir reproductive behaviour in captive settings was found to be affected by the captive environment such as the enclosure type and size, presence of visitors and maternal experience (Arumugam *et al*. 2020). Similarly, the variation in estrus behaviour in this study could be influenced by these factors.

Throughout the study, vulva changes were observed at least once in all females. Signs of swelling include an increase in the labia thickness and elongation of the vulva slit (Fig. 5c). In some tapirs, the dorsal portion of the vulva was bulging and the ventral tip of the vulva was rounded. Additionally, clear, thick and string-like discharge (Fig. 5e) was observed when the vulva size increased in MT2, MT3 and MT5. Similar findings were reported by Schaftenaar *et al*. (2006) and Kusuda *et al*. (2007). MT1 did not have an increase in vulva score from January to February due to an injury to the right labium of the vulva (5 cm × 2 cm deep) sustained from fighting with a male. In an adult tapir, the average length of the vulva slit is approximately 4.8 cm (Lilia *et al*. 2010) and the size could increase during estrus. The length of the slit could be used to indicate swelling of the vulva. However, morphometric measurements of the vulva were not performed in this study to avoid unnecessary stress on the animal and to ensure the safety of the handler. Vulva changes are influenced by the concentration of hormones during different stages of the estrous cycle. During periods of low progesterone concentrations (with high estrogen concentration), the vulva appears swollen and vaginal discharge was observed (Kusuda *et al*. 2007). In this study, based on the increased in vulva score, there was evidence of a cyclical pattern (Fig. 6) with a median interval of 31 days (ranges 28–33 days) between peaks (Table 4). An increase in the vulva score is highly suspected to correlate with the female’s estrus stage: Score 1 = Doubtful; Score 2 = Possibly in estrus; and Scores 3 and 4 = Highly likely to be in estrus. Based on the observation of mounting behaviour in the paired group, which coincided with an increase in vulva score, it is likely that the increase in vulva score in the non-paired group is also an indication of estrus.

Estrus behaviour and increased vulva score was observed in both paired and non-paired tapirs. The estrus behaviours were observed more frequently in the paired group, especially at the time of increased vulva scores which allows us to postulate that this coincides with estrus. Statistically, there is evidence of a significant difference in vocalisation and urine spraying between the paired group and the non-paired group when the vulva score is more than 0.

During the first and second week of March, MT1 had an increase in vulva score which was concurrent with increased mating activity such as trailing, vocalisation and mounting. Subsequently, MT2, MT3, MT4 and MT5 were observed to exhibit increases in their vulva score during the second and third week of March (Fig. 6). During that period, MT2 was also observed to demonstrate an increase in mating activities similar to MT1. Additionally, MT3 displayed an increase in activity such as vocalising, running around the paddock and spraying urine while MT1 was mating. These finding suggest the possibility of a ‘synchronisation of estrus’ as the female could be stimulated by the mating activities (vocalisation and pheromones) of their neighbours. From April onwards, there were no changes in the vulva scores of MT1 and MT2 as both were confirmed to be pregnant through serum progesterone hormone concentration and ultrasonography. Both tapirs also did not exhibit any estrus mating behaviours thereafter. Hence, the rise in the vulva score is likely associated with estrus, making it a suitable non-invasive parameter to determine estrus in captive Malayan tapirs.

## CONCLUSION

In conclusion, relying solely on the changes in estrous behaviour, especially within the non-paired females, does not provide reliable indication for the prediction of estrus. However, the combination of both estrous behaviour and vulva score allows for early prediction of the reproductive status in captive Malayan tapirs. These findings are important to develop a non-invasive method of monitoring the estrous cycle in captive Malayan tapirs.

## Figures and Tables

**Table 1:** Information on the five tapirs used in this study, including their parturition history at the centre.

Group	ID	Age (years)	Birthplace	Parity	Current status of pregnancy	Number of previous offspring
Paired	MT1	18	Captive	Multiparous	Non-pregnant	6
	MT2	5	Wild	Primiparous	Non-pregnant	1
Single	MT3	3	Captive	Nulliparous	Non-pregnant	None
	MT4	> 20	Wild	Nulliparous	Non-pregnant	Unknown
	MT5	> 10	Wild	Nulliparous	Non-pregnant	Unknown

**Table 2:** Ethogram of breeding behaviour of female Malayan tapirs (modified from Gillmore 2007; Kamisan 2017; Suwannaphong *et al*. 2018; Arumugam *et al*. 2020).

Breeding behaviour	Description
Vocalisation	Produce sounds through the oral or sinus cavity.
Sniffing/smelling of excretion	Smelling of male or own excrement (urine or faeces).
Flehmen response	Head elevated and neck extended, the eyes rolled back, the upper lip curled exposing the upper incisors and adjacent gums.
Spraying/squirting urine	Spraying or squirting urine either to the air, to an object, or to another tapir.

**Table 3:** Vulva scoring system in Malayan tapir (modified from Kusuda *et al*. 2007).

Score	Vulva swelling	Score	Vaginal discharge
0	No swelling	0	No discharge
1	Slight swelling	1	Presence of a small amount of clear discharge (seen at the tip of the vulva)
2	Obvious swelling of the whole vulva	2	Presence of a string of clear discharge from the vulva

*Note*: Final vulva score = sum of the vulva swelling score and the vaginal discharge score

**Table 4:** Summary of the event of increase in vulva score of five females Malayan tapir in Sungai Dusun Wildlife Conservation Centre from January to April.

ID	No. of event	No. of event cycle	Duration (days)		Interval length (days)	
	
			Range	Median	Range	Median
MT1	1	0	4–16	9.0	28–33	31.0
MT2	3	2				
MT3	4	3				
MT4	1	0				
MT5	3	2				
Total	12	7				

**Table 5:** Result of the Mann-Whitney U test comparing estrus behaviours between paired group and single group (when vulva score > 0).

Estrus behaviour	Flehmen response	Vocalisation	Urine spraying	Excretion smelling
Mann-Whitney U	7.000	0.000	0.000	4.500
Z	−1.069	−2.566	−2.640	−1.629
Asymp. sig. (2-tailed)	0.285	0.010**	0.008**	0.103

*Note*:

** Correlation is significant at the 0.05 level (2-tailed).

**Table 6:** Result of Spearman’s correlation test between the vulva score (vulva score > 0) and breeding behaviour (flehmen response, vocalisation, urine spray and excretes smelling).

Estrus behaviour	Flehmen	Vocalisation	Urine spray	Excretion smelling
Correlation coefficient	0.900	0.667	0.667	0.975
Sig. (2-tailed)	0.037*	0.219	0.219	0.005*

*Note*:

* Correlation is significant at the 0.05 level (2-tailed).
